# Insights into the Anti-Inflammatory Effects of Soft Tissue Manipulation

**DOI:** 10.3390/biology14101421

**Published:** 2025-10-15

**Authors:** Jonathan W. Lowery, Basil Mustaklem, Connor Wakefield, Hailey Brown, Madeline M. Sasse, Samuel Baule, Sierra Street, Liza Pradhan, Simran Sandhu, Carmela L. Marciano, David C. Eland, Mary Terry Loghmani, Tien-Min Gabriel Chu

**Affiliations:** 1 Wood College of Osteopathic Medicine, Marian University, 3200 Cold Spring Rd., Indianapolis, IN 46222, USA; bmustaklem840@marian.edu (B.M.); cwakefield867@marian.edu (C.W.); hbrown767@marian.edu (H.B.); msasse023@marian.edu (M.M.S.); sbaule843@marian.edu (S.B.); sstreet@marian.edu (S.S.); lpradhan947@marian.edu (L.P.); ssandhu042@marian.edu (S.S.); mella697@gmail.com (C.L.M.); deland@marian.edu (D.C.E.); 2 Division of Academic Affairs, Marian University, 3200 Cold Spring Rd., Indianapolis, IN 46222, USA; 3 Bone & Muscle Research Group, Marian University, 3200 Cold Spring Rd., Indianapolis, IN 46222, USA; 4 Indiana Center for Musculoskeletal Health, School of Medicine, Indiana University, Indianapolis, IN 46202, USA; mloghman@iu.edu (M.T.L.); tgchu@iu.edu (T.-M.G.C.); 5 Indiana Biosciences Research Institute, Indianapolis, IN 46202, USA; 6 Department of Physical Therapy, School of Health and Human Sciences, Indiana University, Indianapolis, IN 46202, USA; 7 Department of Biomedical and Applied Sciences, School of Dentistry, Indiana University, Indianapolis, IN 46202, USA

**Keywords:** inflammation, soft tissue, manipulation, mechanotherapy, muscle, massage

## Abstract

Soft tissue manipulation (STM) is widely used by physical therapists, massage therapists, athletic trainers, and osteopathic physicians to manage musculoskeletal pain, yet its biological mechanisms remain poorly understood. Preclinical studies indicate that STM can alter immune cell behavior in animal models, increasing anti-inflammatory cytokines (IL-4, IL-10) and reducing chemokines such as RANTES/CCL5. Single-cell transcriptomic analyses suggest mechanical treatment may reprogram stromal cells and shift immune cell recruitment in aged or inflamed tissues. However, many of these mechanistic findings have yet to be confirmed in human studies. Early clinical trials show massage therapy can modify circulating leukocytes and reduce cytokine responses, but direct tissue-level evidence in human subjects remains limited. This narrative review summarizes existing insights and emphasizes the need for future clinical investigations targeting populations with chronic inflammation, repetitive-use injuries, post-surgical fibrosis, or age-related muscle decline. We advocate for studies incorporating tissue or fluid sampling, cytokine profiling, and molecular assays such as flow cytometry or transcriptomics to characterize STM’s immunological effects in people. Rather than simply easing symptoms, STM may act as a precision mechanical stimulus that recalibrates immune tone and promotes tissue repair. Bridging basic science with clinical research will be essential to establish STM as a biologically informed, mechanobiology-based therapeutic strategy.

## Simple Summary

Soft tissue manipulation, which includes techniques such as massage and gentle stretching, has long been used to ease muscle soreness and promote recovery. However, the biological reasons for its benefits remain only partly understood. This article brings together research from laboratory studies, animal models, and early human trials to explore how these mechanical forces may calm inflammation and help tissues repair themselves. Evidence suggests that the pressure and movement applied to muscles and connective tissue can influence immune cells, prompting them to shift from producing harmful signals toward those that foster healing. In people, massage has been shown to lower stress hormones and reduce certain inflammatory molecules in the blood. In animals, it can restore muscle strength and improve recovery after injury. Although these findings are promising, more work is needed to confirm exactly how such changes occur in human tissues. By combining biological research with clinical studies, scientists may one day tailor manual therapies as precise, low-risk treatments to reduce chronic inflammation, accelerate recovery, and support healthy aging.

## 1. Introduction

Mechanical forces applied to soft tissues—through interventions such as massage, stretching, or instrument-assisted techniques—have long been reported to alleviate pain and inflammation. However, only recently have controlled studies begun to unravel the biological mechanisms behind these effects. This field of “mechanotherapy” posits that targeted mechanical loading can elicit beneficial cellular and molecular responses in tissues, which may facilitate endogenous repair processes [1,2,3,4]. Additionally, a growing body of evidence indicates that soft tissue manipulation (STM), which is a form of mechanotherapy involving the deliberate application of compressive, shear, or tensile forces to muscles, fascia, or connective tissue using hands or tools, can modulate local and systemic markers of inflammation. Given that excessive or chronic inflammation can impede healing—while a properly regulated inflammatory response is crucial for tissue repair—understanding the mechanistic relationship between applied force and inflammatory modulation could inform more effective therapeutic strategies utilizing this non-invasive, non-pharmacological modality.

This review synthesizes evidence across human studies, in vitro experiments, and animal models to illustrate how mechanical stimuli to soft tissues influence inflammatory pathways. We begin with clinical observations that underscore the relevance of this mechanistic inquiry, then turn to cellular and animal models that offer greater resolution on possible mechanisms. Throughout, we highlight findings that mechanical interventions can both suppress pro-inflammatory factors and promote anti-inflammatory or regenerative responses, depending on variables like the load magnitude, frequency, timing relative to injury, and the biological context (e.g., healthy vs. damaged tissue, young vs. aged), such as those demonstrated in rodent studies where moderate compressive forces (~4.5 N) promoted recovery, whereas higher forces (~7.6 N) induced muscle damage [5]. Dose and timing effects have also been reported in models of disuse and reloading [6,7]. STM is widely used across disciplines—including physical therapy, athletic training, massage therapy, and osteopathic medicine—with many osteopathic manipulative therapy (OMT) techniques (e.g., myofascial release, strain–counterstrain) overlapping conceptually and mechanically with the STM modalities described in this article. A variety of STM approaches exist, and where possible, we highlight technique-specific differences in application and effect. Finally, we discuss how insights from preclinical models might shape the next generation of research in humans.

## 2. Methods

A literature search was conducted using PubMed to identify studies examining the relationship between STM and inflammation. The following search terms were used:

(“soft tissue”) AND (“manipulation” OR “massage” OR “manual therapy” OR “mechanotherapy” OR “osteopathic” OR “osteopathy” OR “OMM” OR “OMT”) AND (“inflammation” OR “inflammatory” OR “cytokine”).

The search included all articles indexed in PubMed through June 30, 2025, and yielded 122 publications, which served as the primary evidentiary basis for this review. This review is a narrative synthesis rather than a formal systematic review, with the aim of integrating mechanistic insights across human, animal, and in vitro studies. We applied no date or language restrictions and studies were first screened at the title/abstract level to determine relevance to STM and inflammation, and those judged irrelevant were excluded. Full texts were reviewed to confirm that interventions involved mechanical loading of soft tissues (manual, instrument-assisted, or mimetic-based) and reported outcomes related to immune or inflammatory markers. Articles were categorized into three groups: (1) human studies, (2) animal models, and (3) in vitro experiments. Risk of bias was not formally assessed given the heterogeneity of study designs, but study quality and mechanistic detail were considered when weighing evidence. Both human and preclinical studies were included, and, for the final set of articles, priority was generally given to primary studies over reviews or other article types. Additional relevant studies were identified through manual citation tracing and/or author expertise to contextualize mechanistic findings and highlight emerging themes. To enhance transparency, we provide Supplemental Table S1 that lists all identified PMIDs, their categorization, and their inclusion/exclusion status.

## 3. Evidence from Human Studies: Mechanical Interventions and Inflammatory Signatures

Several controlled studies in humans have demonstrated that STM can influence systemic and local markers of inflammation. In a clinical trial of 30 healthy young adults, a single session of Swedish massage was associated with significant reductions in serum cortisol and arginine vasopressin (AVP), two hormones known to influence immune function and inflammatory tone—that is, modulating the balance of inflammatory signals toward resolution and repair—within one hour of the treatment [8]. The intervention followed a standardized 45 min Swedish massage protocol delivered by licensed therapists, incorporating effleurage, petrissage, kneading, tapotement, and thumb friction. The control group received light touch using the back of the hand in the same sequence and duration, controlling for therapist interaction, physical contact, and time spent on the table. In the same study, post-massage blood samples also showed decreased levels of pro-inflammatory cytokines such as tumor necrosis factor-alpha (TNF-α) and interleukin (IL)-6, as well as increased levels of lymphocyte activation markers and CD25+ cell counts, suggesting that mechanical input may enhance immune surveillance while dampening inflammatory burden [8]. In a subsequent randomized trial, participants received once-weekly or twice-weekly Swedish massage or light touch sessions over a five-week period. As in the earlier study, both interventions were standardized and delivered by licensed therapists using identical protocols for timing and body regions, with light touch again limited to gentle contact with the back of the hand. This revealed that sustained effects of STM that persist for several days or more differ profoundly depending on the dosage (frequency) of sessions [9]. For example, twice-weekly STM (but not once-weekly) increased serum levels of oxytocin, decreased serum levels of AVP and cortisol, but had minimal effects on lymphocyte markers [9]. Together, these trials were early-phase with relatively small sample sizes and limited follow-up, which constrains generalizability. Replication in larger and more diverse populations will be important to confirm and extend these findings. These limitations highlight the need for subsequent investigations that incorporate direct tissue-level endpoints, such as muscle biopsies or proteomic profiling, to strengthen mechanistic insight.

Subsequent investigations extended these findings. In one randomized study comparing massage and rest in individuals with muscle soreness induced by eccentric exercise, which involves muscle lengthening under load and is commonly associated with delayed-onset muscle soreness, 11 participants underwent unilateral massage with the contralateral leg serving as a non-massaged control. Participants who received massage exhibited ~25% lower IL-6 protein content in muscle biopsy samples taken from the treated limb within 2.5 h of treatment [10]. Massage also reduced TNF-α protein maturation and attenuated NF-κB (p65) nuclear accumulation in the massaged leg, indicating suppression of pro-inflammatory signaling compared with the control leg. These effects were accompanied by 30–40% increases in mitochondrial biogenesis markers (nuclear PGC-1α, COX7B, ND1 mRNA) and were further supported by proteomic analyses showing broad regulation of inflammatory and mitochondrial-related proteins, consistent with a shift away from pro-inflammatory signaling [10].

While most research has focused on healthy volunteers or exercise-induced inflammation, emerging evidence from symptomatic populations offers additional support for STM’s immunomodulatory potential. In a randomized clinical trial involving patients with carpal tunnel syndrome, both traditional massage and the Graston technique (a form of instrument-assisted STM) produced improvements in nerve conduction velocity, wrist strength, and range of motion [11]. These gains were accompanied by significant reductions in pain and enhanced performance on standardized functional status assessments, with effects sustained at three-month follow-up. Although inflammatory markers were not directly measured, the convergence of objective improvements and subjective relief supports the hypothesis that STM may promote tissue recovery and functional restoration through modulation of reparative immune pathways.

Together, these human studies point to a plausible immunomodulatory role for STM, i.e., reducing pro-inflammatory cytokines, dampening HPA-axis activity, and possibly influencing leukocyte behavior and tissue repair dynamics. However, the broader clinical literature reveals mixed findings, with some studies reporting inconsistent or conflicting effects on cytokines such as TNF-α, IL-6, and IL-8, depending on variables like technique, timing, population, and outcome measures [12]. Acknowledging this variability underscores the need for more mechanistically anchored approaches to STM research, including clearer definitions of target biomarkers and context-specific study designs. To visualize the diversity of STM modalities, force applications, and associated inflammatory outcomes, Table 1 summarizes key studies across human, animal, and cellular models. This comparative matrix helps illustrate where effects converge or diverge and where further investigation is needed. The remainder of this review draws from in vitro and animal model systems to explore these underlying mechanisms in more detail.

## 4. Cellular Mechanisms: Insights from In Vitro Studies

In this section, we will describe several lines of evidence from in vitro model systems aimed at delivering defined force profiles to soft tissue cell types. These systems allow for monoculture or co-culture of cells and accommodate molecular-level analyses of gene expression changes and/or cellular responses that may be challenging to observe in vivo.

Particular attention has been paid to fibroblasts, which are connective tissue cells embedded in fascia, muscle epimysium, and dermis, and demonstrate that these cells likely play a critical role in sensing and responding to mechanical stimuli. Once considered passive scaffolding cells, fibroblasts are now recognized as highly dynamic players in tissue remodeling, inflammation, and repair. Their responsiveness to strain and stretch makes them a compelling candidate for mediating the biological effects of STM. In response to mechanical inputs, fibroblasts can change shape, reorganize cytoskeletal structures, activate signaling cascades, and secrete a host of paracrine mediators—including pro- and anti-inflammatory cytokines—that influence surrounding cells. Their behavior in vitro provides key mechanistic insights into how mechanical therapies might regulate inflammation and tissue healing in vivo.

One striking example comes from a novel co-culture model that examined how strained fibroblasts influence nearby muscle cells. In this system, fibroblasts and myoblasts (muscle precursor cells) were grown in adjacent compartments such that only soluble factors could communicate between them, allowing researchers to isolate the biochemical signals released by mechanically stimulated fibroblasts [18]. The type of strain regimen applied to fibroblasts produced markedly different effects in the muscle cells. A cyclic short-duration strain (CSDS)—intended to model repetitive stress or injury—was implemented as smaller static or low-frequency shortening strains maintained for prolonged periods in vitro, and in this context led fibroblasts to secrete factors that disrupted neuromuscular junction development in the adjacent myotubes [19]. These myotubes exhibited hypersensitized contractions in response to acetylcholine and showed loss of normal acetylcholine receptor (AChR) clustering on their surface [18]. Such disorganization mirrors pathological changes observed after muscle denervation or trauma, where AChRs become dispersed and fibers become hyper-excitable. In contrast, when fibroblasts were subjected to an acyclic long-duration strain (ALDS)—higher-magnitude, cyclic lengthening strains applied for shorter intervals in vitro and intended to approximate a therapeutic stretch akin to manual therapy—they secreted factors that promoted larger, more organized AChR clusters and avoided the abnormal contractility [18,19]. These strain regimens are defined by deformation of flexible membranes in culture rather than clinical loading magnitudes, but they provide a reproducible framework for contrasting lengthening versus shortening environments. Taken cautiously, they suggest that the type of mechanical input fibroblasts receive can determine whether they promote a regenerative or disruptive paracrine environment for nearby muscle tissue.

Additional monoculture studies have further detailed the mechanistic basis for these observations. When exposed to a simulated repetitive motion strain (RMS), human fibroblasts show signs of cellular stress and pro-inflammatory activation, develop elongated lamellipodia, lose cell–cell contacts, and undergo increased apoptosis marked by phosphorylation of the pro-apoptotic proteins DAPK and FAK [20,21,22,23,24]. These changes resemble a cellular injury response. However, when a brief stretch mimicking the STM technique of myofascial release (MFR) is applied after the RMS insult, fibroblast morphology normalizes and apoptosis returns toward baseline [22,23]. Importantly, this combination of injurious strain followed by therapeutic stretch elicits changes in cytokine secretion: while RMS alone has limited effect, adding MFR-like stretch leads to significant reductions in pro-inflammatory cytokines including IL-6 and IL-8 in the culture medium [22,24]. These results support a model in which fibroblasts function as mechanosensors capable of shifting between pro- and anti-inflammatory phenotypes depending on the nature of mechanical loading.

Together, these in vitro findings suggest a coherent cellular mechanism: fibroblasts transduce mechanical signals into biochemical cues that can either escalate inflammation and tissue disruption or promote resolution and repair. Although the full complement of mediators is still being identified, fibroblast-derived IL-6 and nitric oxide are consistent features of this response pattern [19,20]. These discoveries lay essential groundwork for interpreting the more complex inflammatory dynamics observed in preclinical and clinical models of STM. At the same time, fibroblast behavior may vary depending on their tissue of origin (e.g., dermal, fascial, muscular, etc.), and findings from one cell type may not fully generalize to others. This variability should be kept in mind when extrapolating in vitro results to whole-tissue or clinical contexts.

## 5. Animal Models: Disuse, Aging, and Recovery

Animal models provide critical insights into the molecular and cellular consequences of STM, particularly in situations where human tissue sampling is not feasible. These studies allow controlled investigation of dose, timing, and force—helping to uncover how mechanical stimuli shape inflammatory and regenerative responses. While clinical evidence demonstrates that STM can improve pain and function, animal research sharpens the lens on why. In many cases, it is not the mechanical force alone that matters, but how that force interacts with age, injury, and immune signaling.

One major line of investigation focuses on disuse-induced skeletal muscle atrophy and the potential for mechanotherapy to accelerate recovery. In rodent models of hindlimb suspension—which mimic muscle unloading during bedrest, prolonged immobility, or spaceflight—moderate cyclic compressive loading has been shown to enhance recovery of muscle mass, stimulate protein synthesis and ribosome turnover, and increase the number of satellite cells [3,4,6,13]. For example, Mustaklem et al. [4] reported that gastrocnemius mass recovered to ~74% of baseline with STM compared to ~50% with re-ambulation alone, with contralateral muscles showing a similar benefit. In this model system, STM applied to atrophic muscle is associated with numerous alterations in cytokines and growth factors in the muscle microenvironment, including ciliary neurotrophic factor (CNTF, ~4.7-fold), matrix metalloproteinase (MMP)-9 (~2.3-fold), resistin (~1.8-fold), IL-2 (~1.9-fold), IL-3 (~1.6-fold), and IL-13 (~1.9-fold) [4]. Interestingly, the effect of STM on muscle recovery is not limited to the treated limb: contralateral muscles also respond, suggesting a systemic signal triggered by local mechanical loading [3,4,7]. Indeed, consistent with the human massage studies referenced above, STM applied to atrophic muscle in rats alters levels of numerous serum factors including G-CSF (~2-fold), IL-13 (~2.4-fold), and TNF-α (~1.8-fold) [4]. These findings reinforce the idea that STM may accelerate recovery not only through anabolic signaling but also by modulating both local and systemic inflammation.

Follow-up studies confirmed that STM is most effective during the reloading phase, after disuse ends. When mechanical loading was applied during recovery, it accelerated muscle regrowth and increased fiber size—a benefit not observed when the same intervention was delivered during disuse [6,7]. These findings suggest that the biological context of loading determines whether molecular signals translate into structural change. In aged rats recovering from atrophy, compressive loading reprogrammed the cellular landscape: single-cell RNA sequencing revealed that stromal and immune cells upregulated chemokines (e.g., Csf1) and matrix remodeling genes (e.g., Col1a1), enhancing the recruitment of immune cells and promoting collagen turnover [14]. Despite age-related impairments in hypertrophic capacity, STM in these animals produced a shift toward a more regenerative, youth-like inflammatory milieu [7,15].

Yet, more is not always better. When higher compressive forces (~7.6 N) were applied to old muscle—in the hope of overcoming blunted mechanosensitivity—the results were counterproductive [5]. Rather than promoting regrowth, the high-load intervention induced muscle damage, evidenced by increased inflammatory infiltrates and regenerating fibers expressing embryonic myosin [5]. A lower load (~4.5 N), though safer, failed to significantly boost recovery. These findings raise the intriguing possibility of a U-shaped relationship, in which insufficient force yields no benefit but excessive force tips into harm. While this interpretation is supported by comparative findings within the same experimental framework, formal analyses spanning a wider range of forces that integrate phenotypic and molecular outcomes have yet to be reported. Moreover, the optimal therapeutic window for STM may be especially narrow in aged or fibrotic tissues [5,7,14,15].

## 6. Animal Models: Repetitive Overuse and Chronic Inflammatory Conditions

Injury models involving repetitive overuse offer another lens through which to examine STM’s immunomodulatory effects. In a rat model of repetitive strain injury, manual therapy delivered concurrently with task performance can prevent the emergence of pathological inflammation. Rats performing a high-repetition forelimb task (standardized at approximately 40–50% of maximal pull using a calibrated force transducer) typically develop CD68^+^ and CD206^+^ macrophage infiltration, collagen deposition, and functional deficits [25,26]. In contrast, animals that were randomly assigned to receive a multimodal STM protocol (skin rolling, muscle mobilization, limb traction), delivered by a trained operator under therapist-developed guidelines and evaluated by blinded outcome assessors, maintained normal sensorimotor function and showed minimal inflammatory or fibrotic remodeling [25,26]. In essence, STM appeared to sustain adaptive remodeling and prevent maladaptive chronic inflammation before it could take hold.

Therapy delivered after injury, while less dramatic, still had measurable benefits. In a follow-up study, rats rested for seven weeks after completing the overuse task—with or without concurrent STM [27]. The therapy group exhibited higher IL-10 levels and improved behavioral recovery compared to rest alone, including reductions in persistent cold hypersensitivity and fibrosis [27]. Although less robust than concurrent treatment, post-injury STM still shifted the tissue environment toward resolution and repair.

A complementary model of chronic low back pain offers further support for STM’s targeted anti-inflammatory effects [16]. In rats with lumbar inflammation induced by Complete Freund’s Adjuvant, instrument-assisted STM applied to the injury site three times weekly for two weeks improves gait patterns and modulates serum levels of cytokines involved in pain and inflammatory response, including increased levels of Neuropeptide-Y (NPY), a neuropeptide proposed to suppress spinal pain signaling and shift microglia toward anti-inflammatory phenotypes [28,29], and reduced levels of RANTES/CCL5, which exerts effects that are generally pro-inflammatory [30]. Additionally, in this model, STM reduced RANTES/CCL5 levels—which is consistent with the changes observed in serum—and increased IL-4 in the paraspinal muscles within two hours of treatment [31]. IL-4 is particularly interesting in this context given that this cytokine has been shown to suppress the production of pro-inflammatory cytokines TNF-α and IL-1β and promote macrophage release of anti-inflammatory factors including IL-1ra and IL-10 [17]. These studies also examined several other relevant cytokines, yet found limited changes, with significant effects largely confined to RANTES/CCL5, IL-4, and NPY within the analytes measured, supporting the interpretation that STM engages specific immunological pathways linked to pain resolution and tissue repair.

Taken together, these animal data illustrate that STM is not a blunt tool—it is a fine-tuned intervention whose effects depend on timing, dose, and biological context. Mechanotherapy can temper maladaptive inflammation, stimulate debris clearance, and support tissue remodeling. But it can also provoke damage if improperly applied. These mechanistic findings complement and clarify what has been observed in humans, deepening our understanding of how therapeutic force interacts with inflammation. As a bridge between clinical efficacy and cellular response, animal models will remain indispensable for identifying optimal parameters and predicting how different patient populations might respond to specific STM techniques.

## 7. Translational Gaps and Future Directions in Human Research

Mechanistic and animal studies provide compelling evidence that STM can modulate inflammation in ways that support tissue repair, immune regulation, and pain resolution. Yet, despite this growing mechanistic clarity, relatively few studies have rigorously evaluated these processes in humans at the cellular or molecular level. Early clinical trials have shown that massage therapy can alter circulating immune cells and dampen ex vivo cytokine release after treatment [8,9], but the specific tissue-level pathways observed in animals—including macrophage phenotype shifts, chemokine modulation, and altered stromal signaling—remain largely unverified in humans.

This translational gap presents a clear opportunity. For example, while rodent models demonstrate that STM increases IL-4 and reduces RANTES/CCL5 in inflamed tissues [31], and shifts immune cell composition in favor of resolution and regeneration [14,26], there are no human studies confirming whether local soft tissue cytokine profiles or immune cell phenotypes change in similar ways after treatment. Likewise, the upregulation of genes involved in matrix remodeling (e.g., Col1a1, Csf1), modulation of growth factors, and cytokines in target tissues, and the recruitment of reparative immune cells seen in aged or atrophied muscle [4,14] has not been replicated in clinical populations such as elderly patients, individuals recovering from disuse, or those with fibrotic soft tissue conditions.

While these specific tissue-level pathways remain largely unverified in humans, there is some evidence that mechanical therapies can influence systemic immune parameters. In one study of healthy adults, visceral manual therapy—using rhythmic compressive techniques to the thorax and abdomen—acutely increased circulating cytokines such as IL-10, IL-8, and G-CSF, and mobilized a subpopulation of inflammatory dendritic cells [32]. Although these visceral approaches differ from musculoskeletal STM in both target tissue and likely mechanism, they support the broader principle that soft tissue manipulation—including techniques applied at a distance from the affected tissue—can modulate immune function beyond the site of application.

Several key translational questions remain unanswered:

- *Local immune changes*
  Can STM induce a shift toward M2-like macrophage phenotypes in human tissues, as suggested by animal studies?
  Are there measurable changes in local cytokines (e.g., IL-4, IL-10, RANTES/CCL5, TNF-α) in human muscle, fascia, or skin following STM?
  Does STM alter stromal gene expression in humans in ways comparable to changes observed in single-cell analyses from rodent models?
- *Systemic vs. local effects*
  Do systemic changes in immune cell subsets (e.g., NK, CD8^+^ T cells) correspond with local tissue responses, or are these distinct phenomena?
  Can systemic hormone or neuropeptide changes (e.g., cortisol, AVP, oxytocin, NPY) observed after massage be directly linked to local tissue repair processes?
  To what extent do contralateral or distant tissue effects (e.g., improvements in untreated limb, bone remodeling) reflect systemic vs. reflexive mechanisms?
- *Individual factors*
  How do age, sex, hormonal status, or comorbid conditions modulate the immunological response to mechanical therapy?
  Does age-related blunting of mechanosensitivity alter the therapeutic window for STM, and can parameters be adjusted accordingly?
  Do baseline inflammatory profiles (e.g., chronic low-grade inflammation in metabolic disease) predict differential responses to STM?

Answering these questions will require studies that go beyond symptom-based outcomes and incorporate direct sampling of blood, tissue, or interstitial fluid, along with techniques such as cytokine arrays, flow cytometry, and single-cell RNA sequencing. Longitudinal designs could clarify whether repeated treatments lead to adaptive immune responses or tissue remodeling, as seen in animal recovery models. Dose-finding studies, guided by insights from force-dependent responses in aged or inflamed tissues, are also essential to define the therapeutic window in different clinical populations.

Importantly, future human studies should move beyond healthy volunteers and target populations most likely to benefit from inflammatory modulation: individuals with overuse injuries, chronic low back pain, post-surgical fibrosis, or age-related muscle decline. These groups mirror the conditions explored in animal models and provide the clearest path for translating basic science into practice. Integrating imaging, biomechanics, and immunology into STM research will allow us to tailor interventions with precision—identifying not only what works, but for whom, how, and why.

Interestingly, STM may also influence distant tissues. In the same repetitive strain model, manual therapy helped preserve bone architecture in overused forelimbs, even as systemic inflammation (e.g., serum TNF-α) remained elevated [33]. This finding suggests that STM might promote adaptive remodeling in bone and muscle alike, even when inflammatory signals are not fully extinguished. Rather than blunting all inflammation, STM may help shape the response—enhancing processes like osteogenesis or matrix reorganization when applied with appropriate timing and load. Two human studies of Thai traditional massage offer further evidence that soft tissue manipulation may modulate bone turnover. Because these trials involved a massage style distinct from the STM modalities emphasized in this review, their results should be interpreted cautiously. Nevertheless, they demonstrate that mechanical inputs to soft tissue can influence systemic processes such as skeletal metabolism. In a crossover trial of 48 postmenopausal women, twice-weekly massage for four weeks significantly increased serum P1NP, a marker of bone formation—particularly among older women with smaller body build [34]. A separate investigation in younger women similarly found that a single two-hour massage acutely increased P1NP and decreased CTx, indicating a shift toward bone formation [35]. This potential ability of mechanotherapy applied to soft tissues to affect skeletal metabolism is also consistent with findings from a rodent model of disuse atrophy, where STM increased levels of osteopontin in both muscle tissue and serum and modestly elevated serum osteoprotegerin [4]. Together, these findings suggest that rhythmic compressive massage may influence osteoblast activity and bone metabolism, even in the absence of high-impact mechanical loading.

## 8. Conclusions

Together, these data suggest that STM has the potential to serve as a mechanically dosed immunomodulator capable of influencing inflammation and tissue repair across multiple biological levels. However, the translational gap between mechanistic findings and routine clinical application remains wide. To bridge this divide, future human studies should incorporate direct molecular endpoints, such as cytokine or growth factor sampling from local tissues before and after treatment. Randomized crossover trials (in which participants receive both STM and a light-touch (or static-touch) control in randomized order, with pre- and post-treatment sampling) would provide a rigorous yet feasible approach for identifying acute mechanistic effects. These designs are particularly practical in small cohorts, since cytokine and gene expression endpoints can be measured after a single treatment session. Parallel-group randomized trials could then extend these insights to longer treatment courses in clinical populations such as tendinopathy or post-surgical fibrosis. Stratification by age, hormonal status, or baseline inflammatory burden may also be critical for revealing differential responses, particularly in populations where tissue repair is impaired. Finally, methods such as microdialysis or biopsy-based immunophenotyping may allow researchers to evaluate tissue-resident immune cell dynamics in situ, adding depth to our understanding of STM’s effects beyond circulating markers. Recent reviews have also highlighted the variability and limited interpretability of cytokine data across manual therapy studies, particularly in the absence of clearly defined immune phenotypes or standardized biomarker panels [12]. This reinforces the need for research that not only measures immune outcomes more precisely but also clarifies which tissue-level responses are truly responsive to STM. By emphasizing these mechanistic targets, the current review aims to support a more biologically grounded framework for future investigation.

As the field of immuno-mechanobiology expands, STM stands poised not merely as an adjunct for symptom relief, but as a biologically targeted intervention that can recalibrate immune tone and regenerative capacity in diverse clinical contexts. These findings reinforce the idea that manual therapies may exert more than palliative effects and, instead, engage specific biological pathways involved in immune regulation and tissue repair. Framing STM as a mechanistically guided intervention opens the door to precision-based treatment strategies in which parameters such as force, frequency, or technique are tailored to an individual’s biological profile—for example, inflammatory biomarker patterns, age-related immune shifts, or comorbid conditions—grounded in measurable physiologic outcomes. By extending mechanistic discoveries into clinical trials with robust biological endpoints, STM may transition from an empiric therapy to a precision-based intervention rooted in immuno-mechanobiology.

## Figures and Tables

**Table 1:** Summary of observed immune and inflammatory effects across soft tissue manipulation (STM) techniques. Arrows indicate reported direction of change in cytokines or immune markers. Abbreviations: AVP, arginine vasopressin; NK, natural killer cells; STM, soft tissue manipulation.

STM Technique	Force Type(s)	Markers Affected	Observed Effects [Citations]
Massage	Moderate to variable compressive (hand-delivered, via instrument, or robotic)	RANTES/CCL5 ↓, IL-6 ↓, IL-8 ↓, TNF-α ↓, IL-4 ↑, IL-10 ↑, CD4^+^ ↑, CD8^+^ ↑, NK ↑, Cortisol ↓, AVP ↓, M2 macrophages ↑	Systemic anti-inflammatory effects and local immune modulation in humans [8,9,10,11] or rodent models [3,4,5,6,7,13,14,15,16,17]
Myofascial Release	Cyclic shear + light compressive	IL-6 ↓, IL-8 ↓, CCL2 ↓, TNF-α ↓	Predominantly in vitro studies suggesting local anti-inflammatory effects via fibroblast mechano-signaling [18,19,20,21,22,23,24]
Cross-Fiber Friction Massage	Transverse compressive/shear	RANTES/CCL5 ↓, IL-4 ↑, CD68^+^ ↓	Reduced macrophage infiltration, fibrosis prevention in rodent models [25,26,27]

## Data Availability

The literature set used for the preparation of this article is available upon reasonable request to jlowery@marian.edu.

## Supplementary Materials

The following supporting information can be downloaded at: https://www.mdpi.com/article/10.3390/biology14101421/s1. Table S1. Comprehensive list of publications identified in the PubMed search examining the relationship between soft tissue manipulation (STM) and inflammation. Each entry includes the PMID, article type (human study, animal model, in vitro experiment, or other), and inclusion/exclusion status for this narrative review. For purposes of categorization, a primary article was defined as an original research study reporting new experimental or clinical data (human, animal, or in vitro). Reviews, meta-analyses, commentaries, and case reports or case series were categorized as “Other”.
